# Supplementary material for: Meiosis-specific decoupling of the pericentromere from the kinetochore
Source: bioRxiv. 2024 Jul 22:2024.07.21.604490. Preprint. [Version 1] doi: 10.1101/2024.07.21.604490 (PMC11291024; doi:10.1101/2024.07.21.604490)

Extended Data Fig. 1

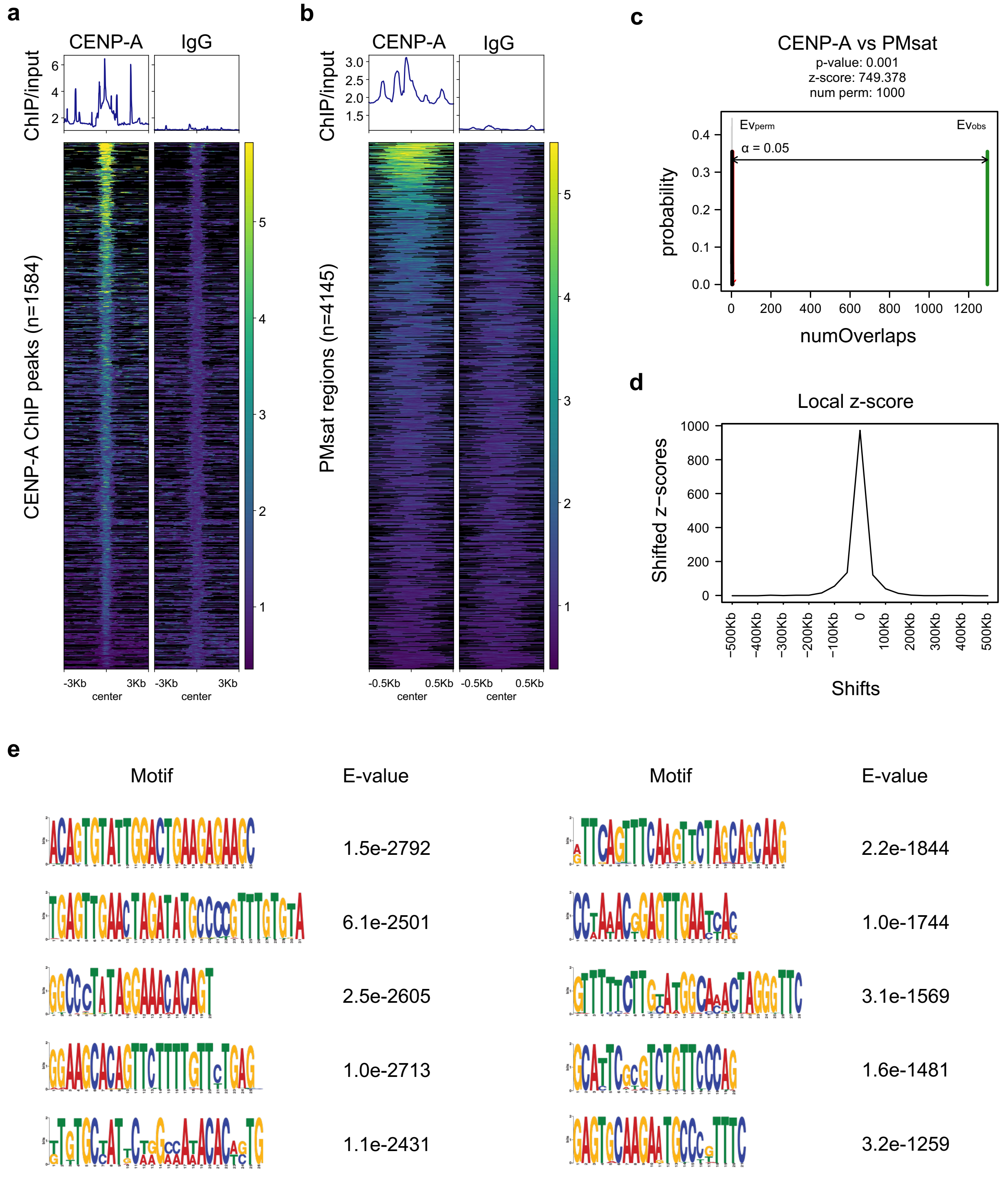

AT**ACAGTGTATTGGACTGAAGAGAAG**CTCCTGTTTTACTtAGTTTCCAATAC**TgAGTTGAACTAGATATGCCCCGTTTGTGTAGGAAGCACA**
GTTCTTTTGTTCTGAGCTAGCTCACTGTACT**GGCCCTATAGGAAACACAGT**A**GAGTGCAAGaGTGCCTGTTTC**TCAAAAGCAGAGT**GTTTTT**
CTTgTAAGGCCGACTGGGGTTCAATACAAcTT**CCTAAACGGAGTTGATTCACATTGAGTTTCAaGTTCTAGCATTAAG**aGTTAATTCAGGGT
AAGAACACCACTA**tTGTGCTATTCTGGCCATAAACAGTG**CAAATA**GCACTCGCGTCTGTTCCCAG**CAA

Extended Data Fig. 2

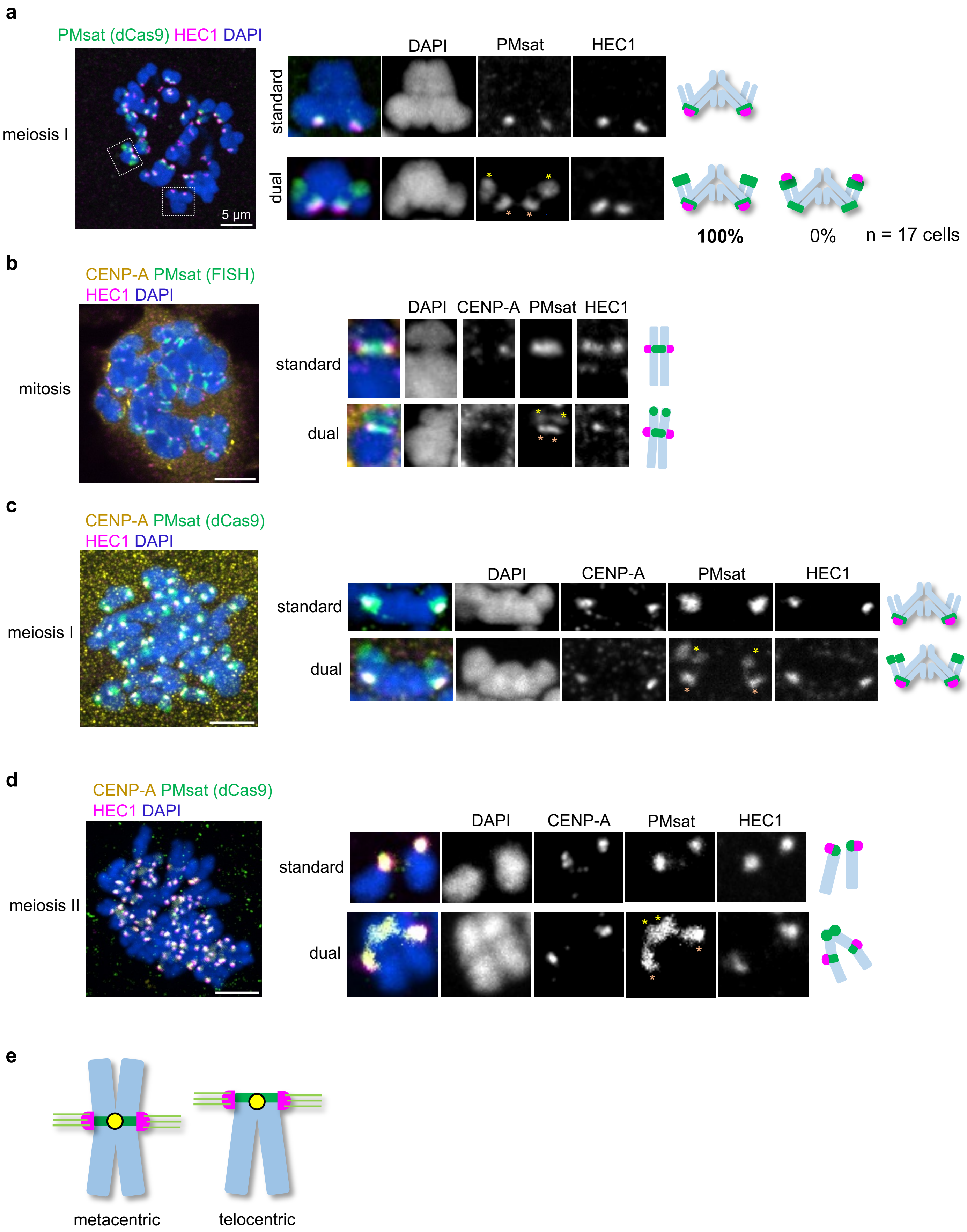

Extended Data Fig. 3

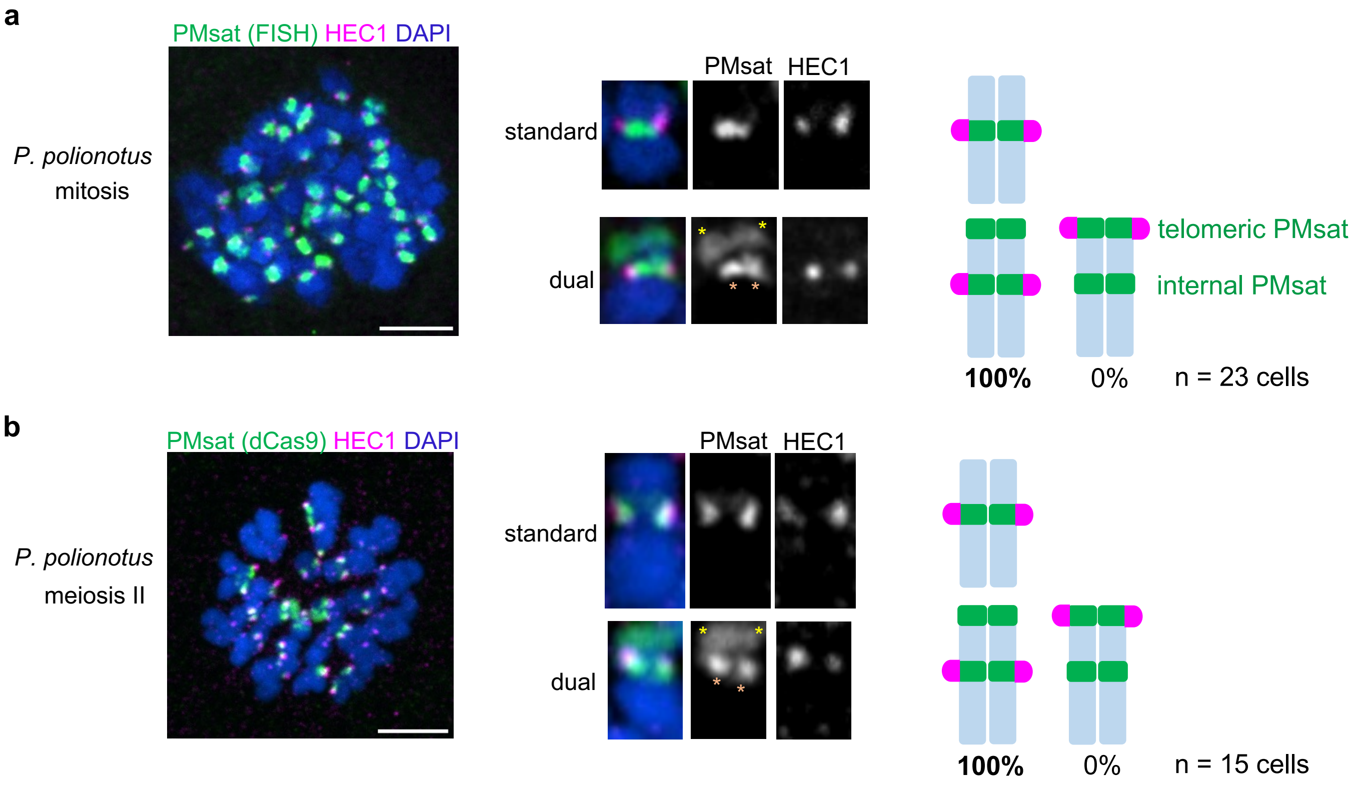

Extended Data Fig. 4

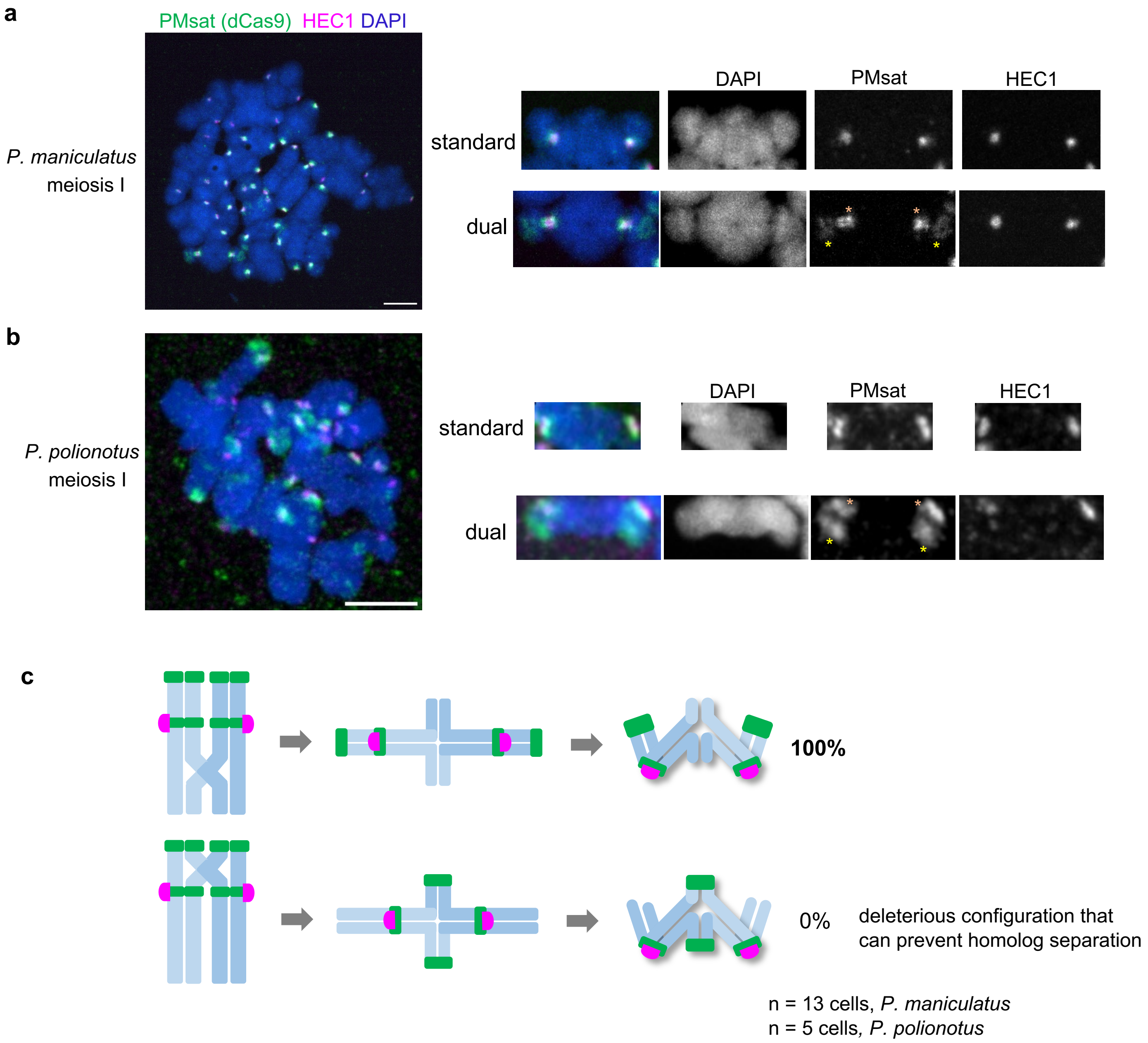

Extended Data Fig. 5

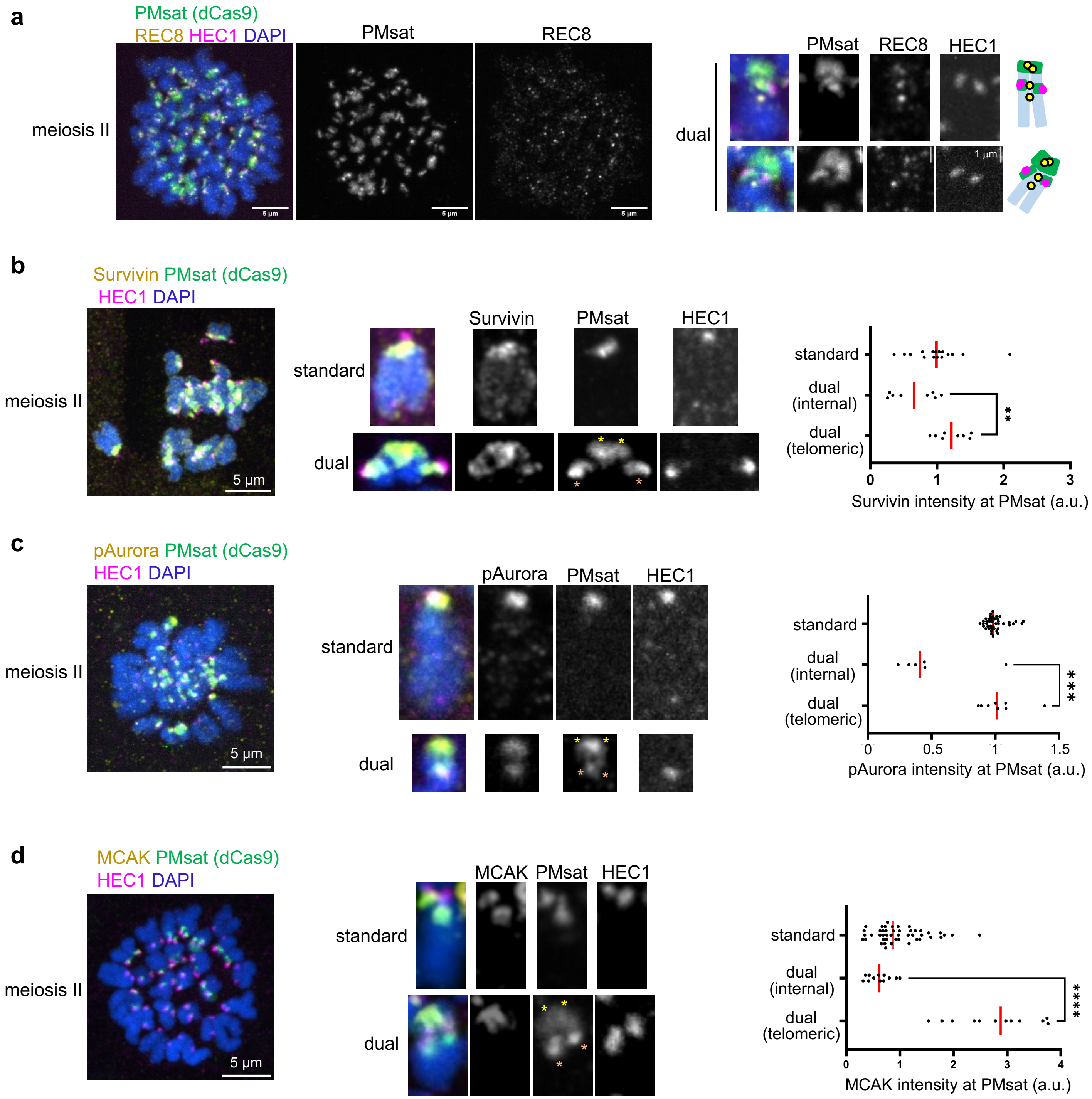

Extended Data Fig. 6

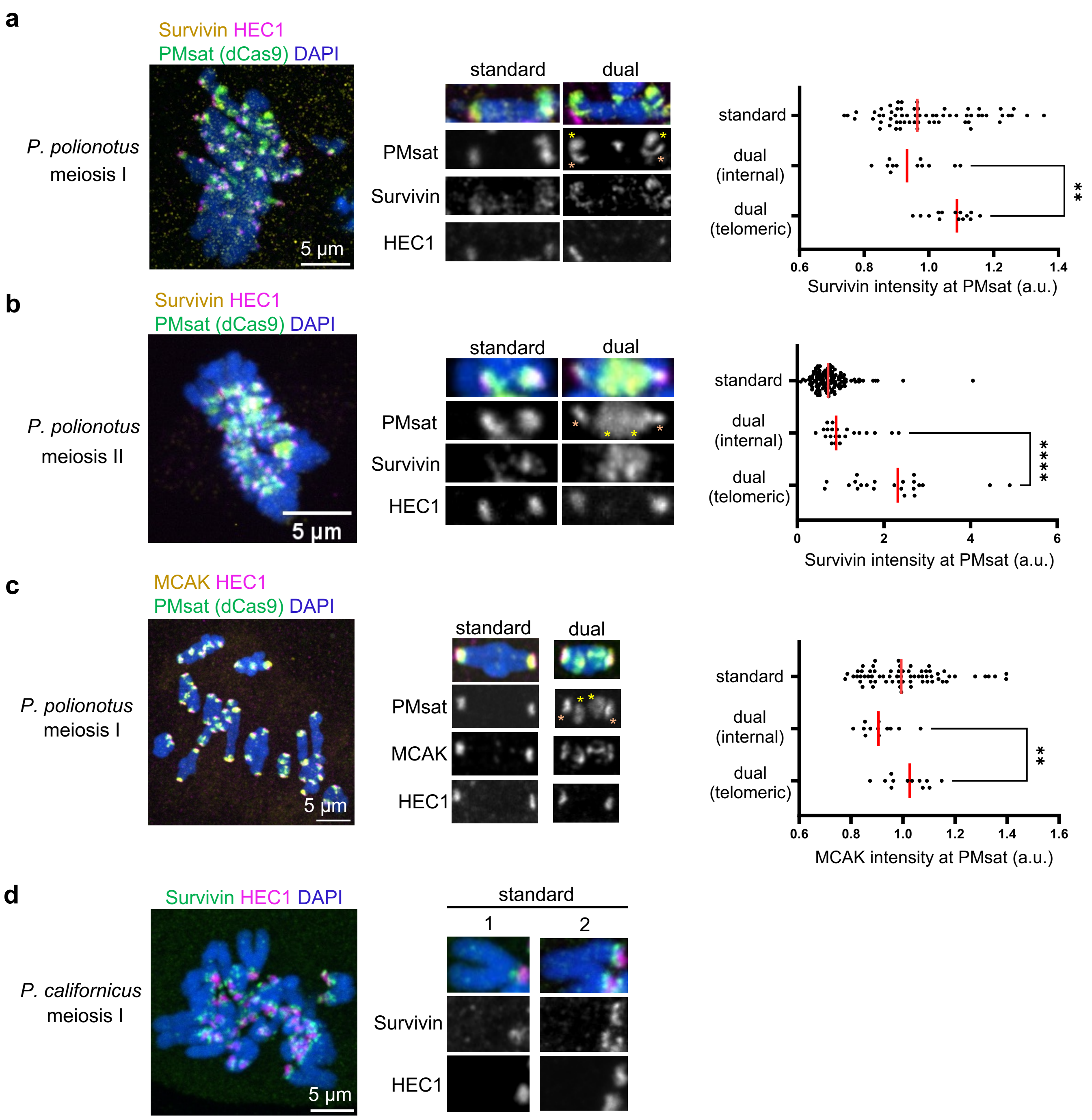

Extended Data Fig. 7

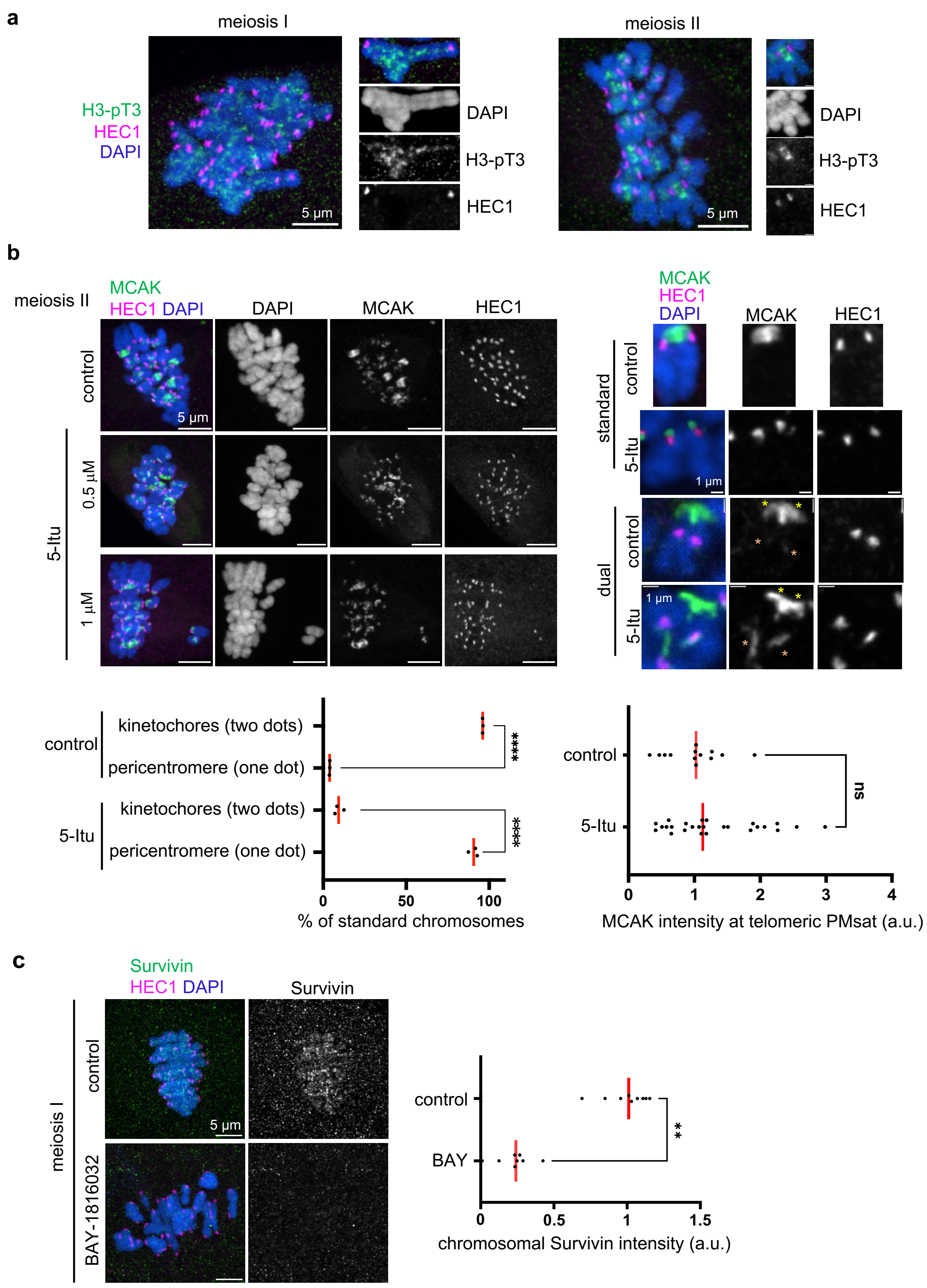

Extended Data Fig. 8

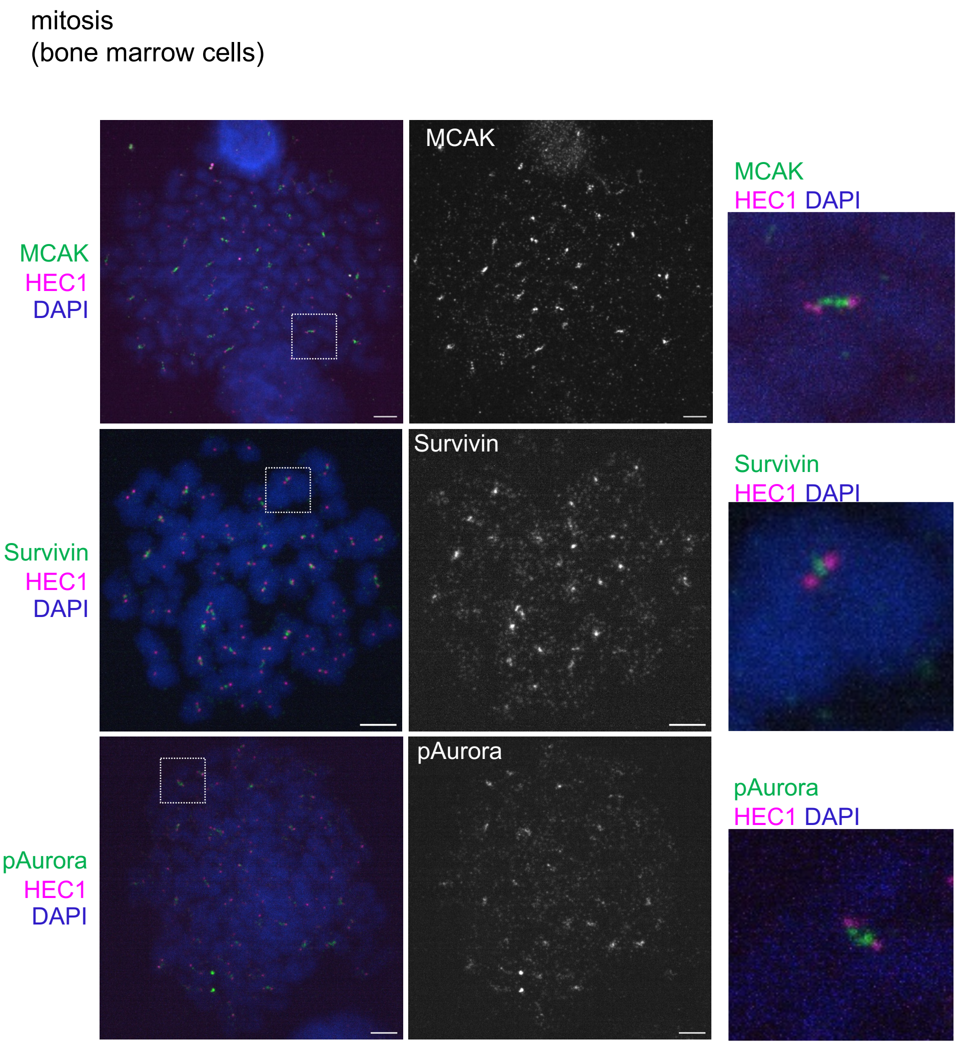

Extended Data Fig. 9

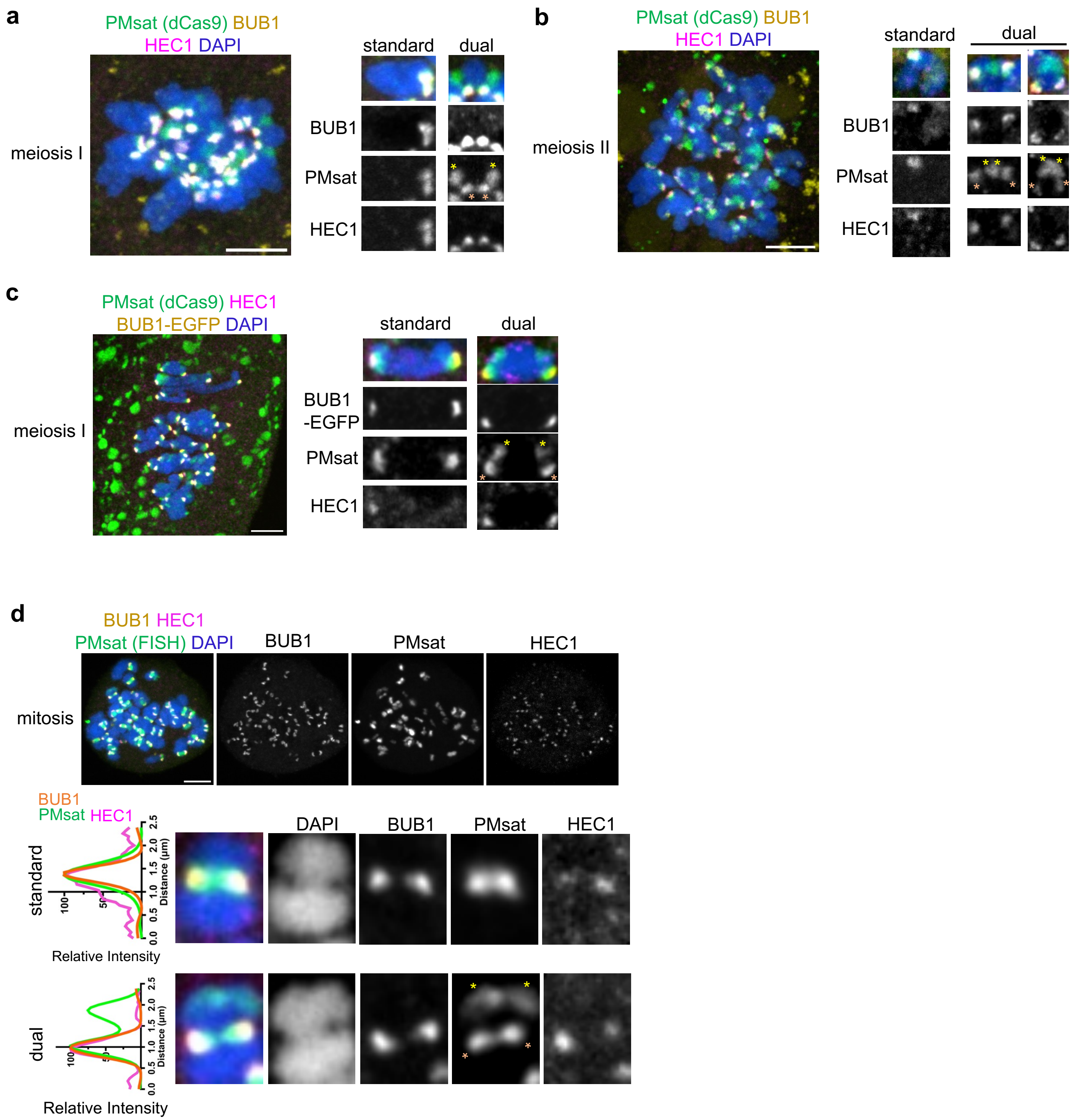

Supplement: Supplement 1 — Extended Data Fig 1. PMsat is the major centromeric DNA in Peromyscus maniculatus. a, Profile plot and heatmap of CENP-A and IgG signal (ratio over input) at CENP-A peaks. b, Profile plot and heatmap of CENP-A and IgG signal (ratio over input) at PMsat regions. c, Representation of the permutation test results to assess association between CENP-A enriched regions and PMsat regions. The number of overlaps was used as evaluation function. The association is highly significant, as the observed value (EVobs) is very distant from the mean of number of overlaps with randomized regions (EVperm) and from the limit of significance of the random distribution (red line). d, Local Z-score plot showing that the association between CENP-A and PMsat regions is highly dependent on their exact position. e, Top 10 de novo identified motifs enriched at CENP-A peaks. The motifs all overlap with portions of the PMsat consensus sequence shown at the bottom. The portions represented by the motifs are highlighted by underlines. Extended Data Fig 2. Kinetochores assemble at internal PMsat in both mitosis and meiosis in Peromyscus maniculatus. a, P. maniculatus meiosis I oocytes expressing dCas9-EGFP and gRNA targeting PMsat were fixed and stained for HEC1. The proportion of chromosomes that assemble kinetochores at internal PMsat and telomeric PMsat was quantified; n = 17 cells from three independent experiments were examined. b-d, P. maniculatus mitotic cells (b), meiosis I (c), and meiosis II oocytes expressing dCas9-EGFP with gRNA targeting PMsat (d) were fixed and stained for CENP-A. n = 11, 12, and 15 cells from three independent experiments were analyzed for mitosis, meiosis I, and meiosis II, respectively. The images are maximum projections showing all the chromosomes (left) and optical sections to show individual chromosomes (right); asterisks denote the chromosomal location of internal PMsat (orange) and telomeric PMsat (yellow) on dual PMsat chromosomes; scale bars, 5 µm. e, Schem [file media-1.pdf]
